# Molecular Mechanisms of Human Disease Mediated by Oncogenic and Primary Immunodeficiency Mutations in Class IA Phosphoinositide 3-Kinases

**DOI:** 10.3389/fimmu.2018.00575

**Published:** 2018-03-19

**Authors:** Gillian L. Dornan, John E. Burke

**Affiliations:** ^1^Department of Biochemistry and Microbiology, University of Victoria, Victoria, BC, Canada

**Keywords:** primary immunodeficiency, oncogenic mutations, phosphoinositides, phosphoinositide 3-kinase, PIK3R2, PIK3R1, PIK3CA, PIK3CD

## Abstract

The signaling lipid phosphatidylinositol 3,4,5, trisphosphate (PIP_3_) is an essential mediator of many vital cellular processes, including growth, survival, and metabolism. PIP_3_ is generated through the action of the class I phosphoinositide 3-kinases (PI3K), and their activity is tightly controlled through interactions with regulatory proteins and activating stimuli. The class IA PI3Ks are composed of three distinct p110 catalytic subunits (p110α, p110β, and p110δ), and they play different roles in specific tissues due to disparities in both expression and engagement downstream of cell-surface receptors. Disruption of PI3K regulation is a frequent driver of numerous human diseases. Activating mutations in the *PIK3CA* gene encoding the p110α catalytic subunit of class IA PI3K are frequently mutated in several cancer types, and mutations in the *PIK3CD* gene encoding the p110δ catalytic subunit have been identified in primary immunodeficiency patients. All class IA p110 subunits interact with p85 regulatory subunits, and mutations/deletions in different p85 regulatory subunits have been identified in both cancer and primary immunodeficiencies. In this review, we will summarize our current understanding for the molecular basis of how class IA PI3K catalytic activity is regulated by p85 regulatory subunits, and how activating mutations in the PI3K catalytic subunits *PIK3CA* and *PIK3CD* (p110α, p110δ) and regulatory subunits *PIK3R1* (p85α) mediate PI3K activation and human disease.

## Introduction

Phosphoinositide 3-kinases (PI3Ks) are essential mediators of signaling downstream of cell-surface receptors and play essential roles in numerous cellular processes, including growth, metabolism, and differentiation (1). PI3Ks generate the lipid phosphatidylinositol 3,4,5, trisphosphate (PIP_3_), which recruits signaling proteins containing PIP_3_ binding domains. Many signaling proteins are activated by PIP_3_, including AGC family Ser/Thr kinases, TEK family tyrosine kinases, and modulators of Ras superfamily GTPases, specifically Guanine nucleotide exchange factors (GEFs), and GTPase activating proteins (2). One of the most well studied PIP_3_ effectors is the AGC protein kinase Akt, which plays key roles in regulating growth and metabolism (3).

The class IA PI3Ks are composed of three p110 catalytic subunits (p110α, p110β, and p110δ), which form an obligate constitutive heterodimer (4) with one of five p85-like regulatory subunits (p85α, p85β p55α, p50α, and p55γ). Class IA PI3Ks are activated downstream of receptor tyrosine kinases (RTKs) and other tyrosine phosphorylated receptors/adaptors, G-protein-coupled receptors (GPCRs), and Ras superfamily GTPases. The p110α and p110β catalytic subunits are ubiquitously expressed, while the p110δ and p110γ subunits share a more restricted immune cell-specific expression profile (5). Knockin genetic models and isoform-selective inhibitors have revealed the essential roles of specific PI3K isoforms, including p110α in insulin and growth factor signaling (6, 7), and p110δ and p110γ in mediating immune cell development and function (8–11).

Due to this fundamental role in a plethora of vital functions, the misregulation of PI3K signaling occurs in various human diseases (2). Underlying the importance of maintaining regulated levels of PI3K activity, disease can be caused by overactive and inactive PI3K signaling. The first clinically approved therapeutic Idelalisib specifically targeting p110δ was FDA approved in 2014 and has shown efficacy in the treatment of B cell-related malignancies (12–16). Even though p110δ inhibitors have shown promise as therapeutics, careful consideration of unexpected complications is critical, as long-term inhibition of p110δ signaling can lead to B cell genomic instability through an Activation-induced cytidine deaminase (AID)-dependent mechanism (17).

Mutations in both catalytic and regulatory subunits frequently activate lipid kinase activity through modification/disruption of inhibitory interfaces between the two subunits. Fundamental to understanding how mutations in different catalytic and regulatory subunits modify PI3K signaling in different cells/tissues is understanding how class IA PI3Ks are regulated by their p85 regulatory subunits, and how they are activated downstream of different activating stimuli. This review will specifically focus on the molecular mechanisms of how class IA PI3Ks are regulated, and how both oncogenic and primary immunodeficiency mutations/deletions in catalytic and regulatory subunits lead to disease.

## Class IA PI3K Regulation

The class IA regulatory subunits have three key roles: they stabilize the p110 catalytic subunit, they inhibit p110 catalytic activity, and they allow for the activation of activity downstream of proteins containing phosphorylated YXXM motifs through engagement of p85 SH2 domains (18, 19). While class IA catalytic subunits require a regulatory subunit for stability, the p85 subunits have been postulated to exist alone and can mediate cellular functions free of p110 (20, 21).

**Figure 1:**
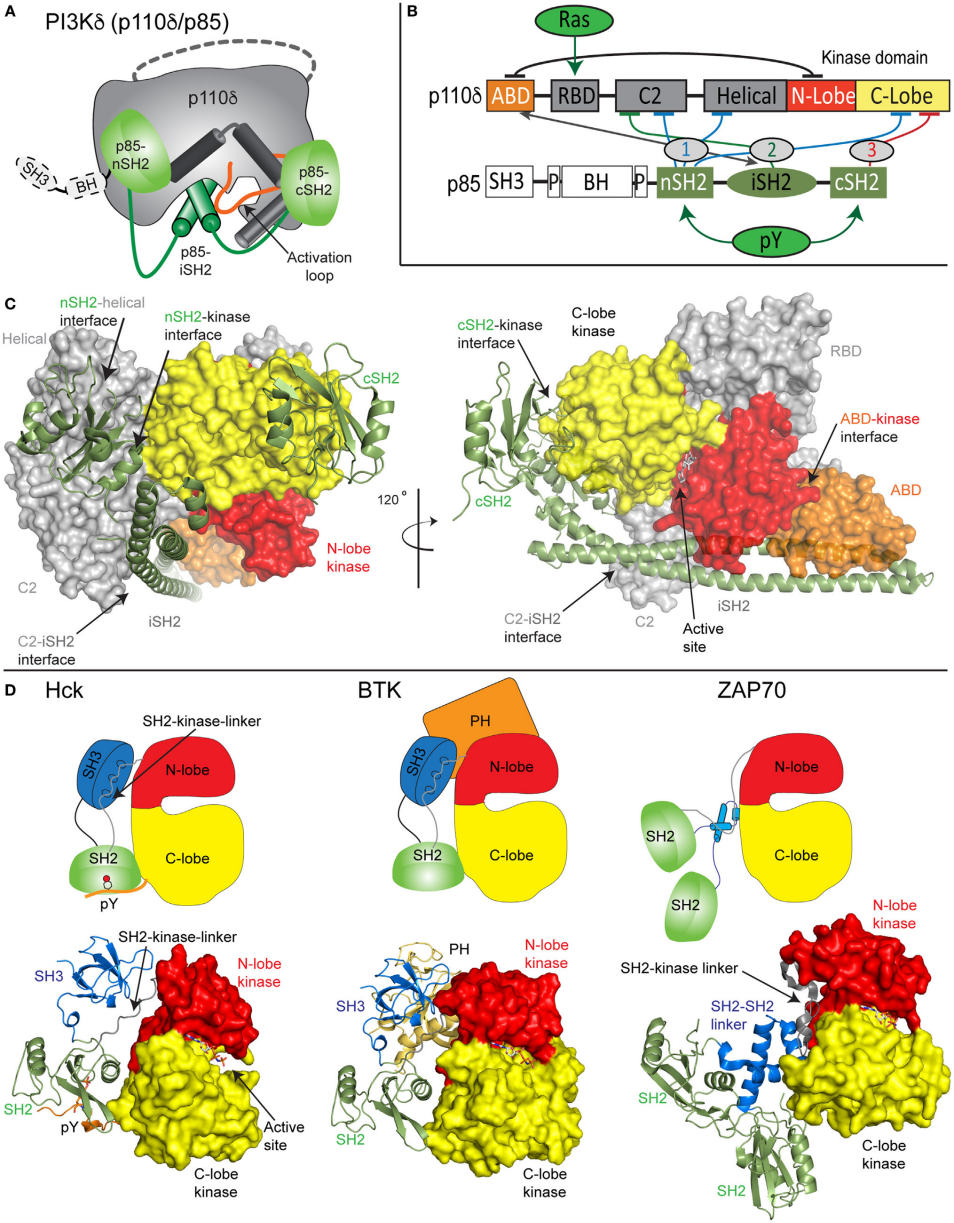
Model of class IA phosphoinositide 3-kinase (PI3K) complex of p110δ/p85α and comparison with other SH2-regulated kinases. **(A)** Cartoon model of the complex of p110δ/p85α, with key features annotated. **(B)** Domain schematic of p110δ and p85α with binding interfaces indicated by the double-sided arrow and inhibitory interfaces indicated by the numbered lines. Activators of PI3K [Ras and phosphorylated receptors (pY)], and their sites of interaction are indicated. The cSH2 domain of p85 only inhibits the p110β and p110δ isoforms and does not inhibit p110α. **(C)** Structural model of p110δ/p85α generated from multiple structures (23–25). The domains are colored according to the scheme shown in panel **(B)**. The p110 catalytic subunit is shown as a surface, and the p85 regulatory subunit shown in cartoon representation. Inhibitory intra- and inter-domain interfaces are annotated, and an inhibitor bound in the active site is shown in sticks. **(D)** Structures of the inhibited forms of SH2-regulated protein kinases involved in immune signaling, Hck (26), BTK (27), and ZAP70 (28) are shown along with cartoon representations indicating how SH2 domains inhibit kinase activity. This shows the various mechanisms of how SH2 domains can inhibit kinase activity, and the key differences in how p85 SH2 domains inhibit PI3K lipid kinase activity.

Both the class IA PI3K p110 catalytic subunit and p85 regulatory subunit are large, dynamic multi-domain proteins (Figures 1A–C). p110 is composed of an adaptor-binding domain (ABD), which interacts with p85, a Ras-binding domain (RBD), which mediates interaction with Ras superfamily GTPases, a C2 domain, a helical domain, and a bi-lobed kinase domain, composed of an N-lobe and a C-lobe. All class IA regulatory subunits contain two Src homology 2 domains [referred to as nSH2 and cSH2 to denote N-terminal and C-terminal] connected by a coiled-coil domain known as the inter SH2 (iSH2). The main interface holding the PI3K heterodimer together is the very tight interaction of the ABD of p110 with the iSH2 domain of p85 (22). Both p85α and p85β subunits also contain a Src homology 3 domain (SH3) and a bar cluster region homology domain (BH). A comparison of class IA PI3K domain organization compared with other SH2 containing protein kinases including Src family and Syk family kinases reveals the large size and complexity of the p110/p85 complex relative to other signaling kinases (Figures 1C,D).

Biochemical/biophysical studies have informed the molecular mechanism of how regulatory subunits bind and inhibit the different p110 catalytic subunits (18, 19, 22, 24, 25, 29–35). A number of inter- and intra-subunit interactions mediate inhibition of each of the class IA catalytic subunits (annotated on the domain schematic in Figure 1B). In all class IA PI3Ks, the ABD domain forms an intra-subunit inhibitory contact with the N-lobe of the kinase domain (32). How the ABD interacts with kinase domain is mediated by the ABD–RBD linker, which packs against the ABD. The C2 domain of p110 forms an inhibitory contact with the iSH2 domain of p85 regulatory subunits. Intriguingly, different p110 subunits have diverse capabilities to be inhibited by this interaction, with p110β being less inhibited by the C2–iSH2 interaction (36), compared with p110α and p110δ.

The nSH2 forms inhibitory interactions with the C2, helical, and C-lobe of all class IA p110 catalytic subunits (22, 24, 29, 30). The C-terminal SH2 domain, which interacts with the C-lobe of the kinase domain, only inhibits p110β (25) and p110δ (30). This interaction cannot occur in p110α due to a loop extension that sterically prevents this inhibitory interaction. Intriguingly, the nSH2 and cSH2 domains have different inhibitory interfaces with p110, with the nSH2 interacting with p110 through its pY binding site, and the cSH2-p110 interface not directly involving the pY binding site. Upon interaction with pYXXM motifs in phosphorylated receptors and their adaptors, the nSH2 and cSH2 interfaces with p110 are disrupted. Different regulation of class IA PI3Ks by their regulatory subunits has important functional implications for how they can be activated by different activating stimuli.

## Signaling Inputs

The ability of PI3K isoforms to mediate signaling in different tissues is a balance between differential PI3K expression and their unique ability to be activated by GPCRs, Ras superfamily GTPases, and phosphorylated receptors/adaptors. All class IA isoforms can be activated by proteins containing phosphorylated YXXM motifs, as this leads to SH2-mediated recruitment of regulatory subunits, and disruption of SH2 inhibitory contacts (22, 29, 30, 35) with the p110 catalytic subunits. p110α is more sensitive to activation downstream of a phosphopeptide derived from platelet-derived growth factor receptor than either p110β or p110δ *in vitro* (29), and this is likely due to the absence of the cSH2 inhibitory interface, which makes the cSH2 more accessible to interact with pYXXM motifs. *In vivo* evidence in support of free SH2 domains being more available to pYXXM motifs is that the E545K mutant of p110α, which disrupts the nSH2–helical interface (described in the following section), is more readily recruited to phosphorylated insulin receptor substrate proteins (37).

Class IA PI3Ks are activated downstream of the Ras superfamily of GTPases through interactions with the RBD domain present in p110 catalytic subunits (38, 39). The Ras superfamily is large and diverse, composed of five main families (Ras, Rho, Rab, Ran, and Arf) (40). The PI3K isoforms are differentially activated downstream of Ras superfamily members (39, 41), with p110α and p110δ being activated downstream of Ras family GTPases, and p110β being activated downstream of Rho family GTPases. Ras activates PI3K through enhanced membrane interaction, with Ras activation being strongly synergistic with activation downstream of phosphorylated receptors (42, 43). Mutant p110α deficient in its ability to be activated by Ras leads to decreased oncogenic transformation, tumor maintenance, and angiogenesis downstream of mutant Ras (44–46).

Class IA PI3Ks can synergize direct and indirect inputs downstream of specific upstream stimuli. p110β is unique in being activated downstream of phosphorylated receptors/adaptors, GPCRs, and Rho family GTPases (47). The ability of p110β to integrate signals from RTKs and GPCRs is critical in its signaling role in myeloid cells (48). p110α is sensitive to activation downstream of insulin receptors due to it being both directly and indirectly activated through RTK-mediated activation of Ras. The ability of different isoforms to be activated downstream of different upstream stimuli plays a key role in determining the capability for activating somatic point mutations to mediate human disease.

## Mutations of *PIK3CA, PIK3CD*, and *PIK3R1* in Cancer, Developmental Disorders, and Primary Immunodeficiencies

### Class IA PI3Ks in Cancer and Developmental Disorders

**Figure 2:**
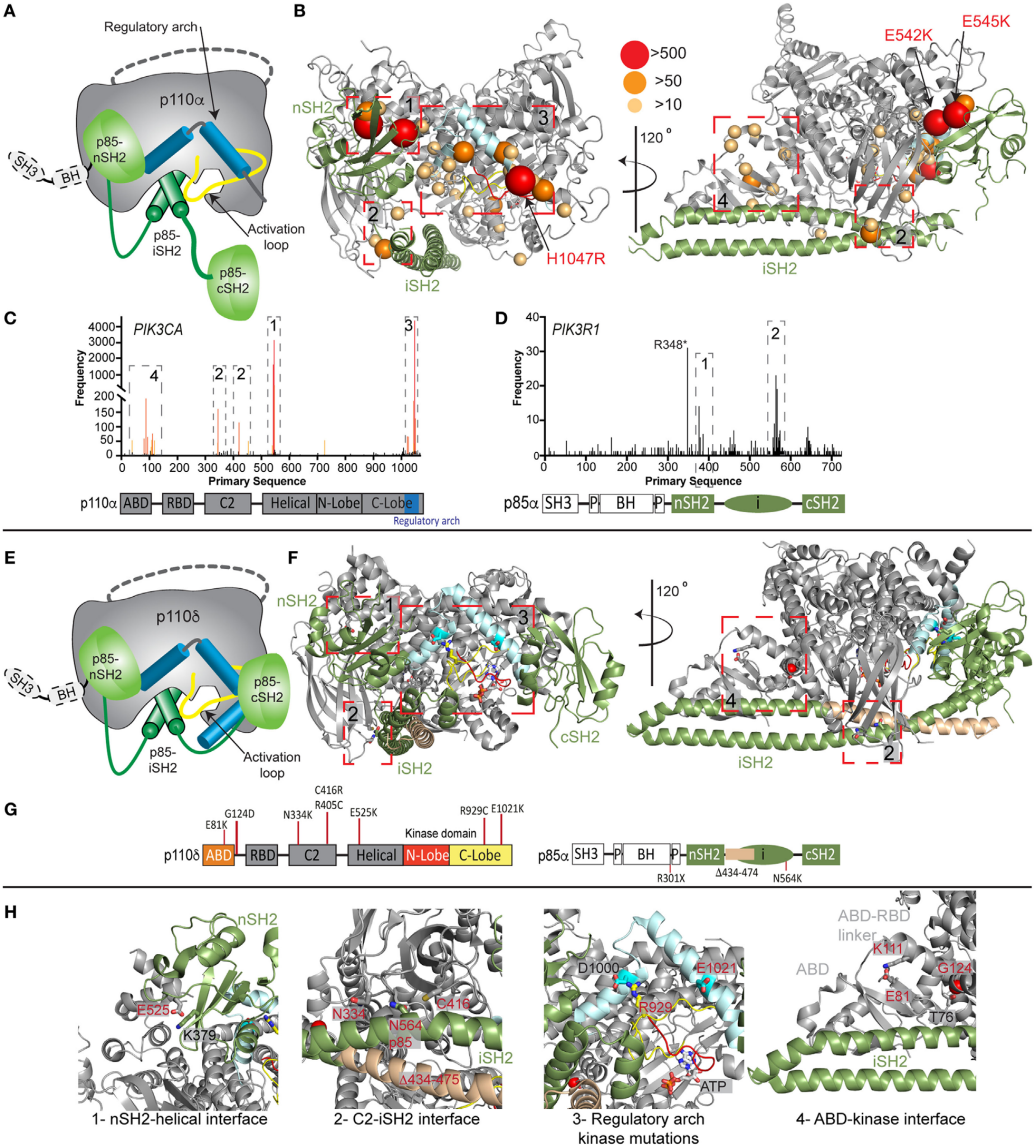
Oncogenic and primary immunodeficiency mutations in *PIK3CA, PIK3CD*, and *PIK3R1*. **(A)** Cartoon schematic of the complex between p110α and p85α with key regulatory features annotated. **(B)** The locations of oncogenic mutations in *PIK3CA* are shown on a structural model of p110α and p85α (24), with the frequency of mutations annotated according to the legend [frequency derived from the Catalogue of Somatic Mutations in Cancer (COSMIC), http://cancer.sanger.ac.uk/cosmic]. The proteins are colored according to the cartoon in panel **(A)**. Regulatory interfaces [N-terminal SH2 domain (nSH2)–helical, C2–inter SH2 (iSH2), regulatory arch, and adaptor binding domain (ABD)–kinase] are boxed and numbered. Boxed regions 1–4 represent mutation hotspots in key regulatory regions. These are enlarged in panel **(H)** in the context of patient mutations in p110δ and p85α. **(C,D)** Frequency of somatic mutations in *PIK3CA* and *PIK3R1* shown on the primary sequence, with the domain schematic indicated below. The locations boxed on the structure in panel **(B)** are also indicated on primary sequence. **(E)** Cartoon schematic of the complex between p110δ and p85α with key regulatory features annotated. **(F)** The locations of primary immunodeficiency mutations in *PIK3R1* are shown on a structural model of p110δ and p85α (23). Boxed regions 1–4 represent mutation hotspots in key regulatory regions. These are enlarged in panel **(H)** in the context of patient mutations in p110δ and p85α. **(G)** Domain schematic of p110δ and p85α with locations of immune-linked mutations in *PIK3CD* and *PIK3R1* indicated. **(H)** Zoom in on molecular details of activating phosphoinositide 3-kinase (PI3K) delta syndrome mutations in p110δ and p85α, focused on the regulatory interfaces boxed in panel **(F)**, with all mutated residues and their interacting residues shown as sticks.

The importance of PI3K activity being properly regulated in human health is underscored by a vast array of human diseases caused by mutations in class IA PI3Ks (mutations in class I PI3Ks in immune disorders and developmental disorders are summarized in Table S1 in Supplementary Material). Mutations can arise in the germline *de novo* or be inherited in an autosomal dominant or recessive manner, and can also arise somatically in specific tissues. Somatic point mutation frequency in cancer in both *PIK3CA* (49) and *PIK3R1* (20, 50) is indicated in Figures 2C,D. Intriguingly, *de novo* germline and postzygotic, somatic mosaic mutations in similar locations in *PIK3CA* and *PIK3R2* (p85β) also lead to overgrowth and developmental disorder syndromes (51–56), revealing that the same mutant can lead to cancer and/or developmental disorders. There are two hotspot regions in *PIK3CA* located at the nSH2–helical interface (E542K and E545K) and the C-terminus of the kinase domain (H1047R) involved in membrane binding (Figures 2B,C). However, in addition, there are numerous rare mutations distributed throughout the primary sequence, primarily localized at the ABD–kinase interface, ABD–RBD linker, C2–iSH2 interface, and the regulatory arch of the kinase domain which is situated over the active site (Figures 2A,B). Rare mutations activate lipid kinase activity, induce oncogenic transformation (31, 57, 58), and are found in endometrial cancers (59).

Mutants located at the ABD–kinase, C2–iSH2, and nSH2–helical interfaces activate lipid kinase activity through disruption of these inhibitory contacts. Intriguingly, there appears to be allosteric long range coupling between these sites, as disruption of the C2–iSH2 interface also leads to disruption of the ABD–kinase interface (31). Mutations within the regulatory arch (a region composed of the two most C-terminal helices, kα10 and kα11, residues 1017–1049) appear to work through a separate mechanism, where conformational changes induced by these mutations drive increased membrane recruitment (31, 60). The regulatory arch lies directly over the active site of the enzyme (Figure 2A). Different mutations induce oncogenic transformation through different mechanisms, with the H1047R mutant requiring p85-mediated recruitment to RTKs, and no longer requiring Ras for transformation, while the E545K mutation still requires input from Ras, and no longer requires p85-mediated RTK activation (58). This is consistent with the putative mechanism of Ras activation, where Ras drives membrane recruitment, and H1047R evades this requirement due to enhanced membrane binding (42, 43).

Somatic cancer-associated point mutations in *PIK3R1* are similarly localized at regulatory interfaces (Figures 2B–D), with the most frequent mutation occurring at the C2–iSH2 interface (N564K/D). These mutants primarily activate PI3K signaling through p110α activation (50, 61, 62). Loss of p85α is also a driver of cancer as it acts as a tumor suppressor, and oncogenic transformation due to loss of p85α is also driven by p110α (63). Several deletions/truncations identified in *PIK3R1* also can mediate oncogenic transformation through different mechanisms. Truncations at the C-terminus of the iSH2 domain can still interact with p110 subunits and disrupt inhibitory contacts (62), leading to increased PI3K activity. Intriguingly, oncogenic truncations also occur N-terminal to the iSH2 domain, and they are unable to bind p110 subunits. These truncations are proposed to function through modification of free p85 interactions with binding partners (20, 21, 64), including the antagonist of PI3K signaling, the phosphatase PTEN.

Mutations in *PIK3R1* leading to decreased PI3K signaling are also found in patients with developmental disorders, with autosomal-dominant or *de novo* mutations in the cSH2 (R649W, K653*, and Y657*) leading to insulin resistance, and dramatically decreased PI3K signaling (65–71). This condition is defined as SHORT syndrome (Short stature, hyperextensibility of joints and/or inguinal hernia, ocular depression, Rieger anomaly, and teething delay) and is caused by the inability of the cSH2 domain to interact with phosphorylated RTKs, as mutation of R649 disrupts the FLVR motif critical for SH2 binding to phosphorylated pYXXM motifs.

### Class IA PI3Ks in Primary Immunodeficiencies

Activating, autosomal-dominant and *de novo* mutations in *PIK3CD* and *PIK3R1* have been discovered in patients with primary immunodeficiencies, and this condition is called activating PI3K delta syndrome (APDS), which is also referred to as PASLI (p110 delta activating mutation causing senescent T cells, lymphadenopathy, and immunodeficiency). Mutations in *PIK3CD*, referred to as APDS1, are found in similar locations to oncogenic mutations in p110α, with mutations discovered at the ABD (E81K), ABD–RBD linker (G124D), C2–iSH2 interface (N334K, R405C, and C416R), nSH2–helical interface (E525K and E525A), and at the C-terminus of the kinase domain (R929C, E1021K, and E1025G) (Figures 2E–H) (72–86). Biochemical experiments have revealed, similar to p110α mutations, that activation occurs due to disruption of p85-mediated regulatory inputs and conformational changes that promote membrane binding (83, 87). The most prevalent mutation in APDS1 is E1021K (similar location to H1047R in p110α); however, APDS mutations in p110δ are more frequently found distributed throughout the primary sequence compared with p110α (Figures 2C,D,G). In line with this observation, E1021K leads to a smaller increase in p110δ lipid kinase activity compared with H1047R p110α. It is likely that additional mutations in *PIK3CD* will be discovered that mimic previously discovered oncogenic mutations in *PIK3CA*, highlighting the need to sequence the entire *PIK3CD* gene in patients presenting with complex immunodeficiencies.

Mutations in *PIK3R1*, referred to as APDS2, have also been identified in a number of immunodeficiency patients, with the most frequent mutation resulting in a splice variant that removes exon 11 (resulting in a p85α with region 434–475 deleted, located at the N-terminus of the iSH2 domain) (88–92). *In vitro*, this deletion leads to increased activation of p110δ compared with p110α, and this is mediated through disruption of all p85 regulatory inputs for p110δ, and only partial disruption of p85 regulatory inputs for p110α (87). This mutant may decrease protein stability of p110 subunits, and there have been reports of these patients having symptoms consistent with both SHORT syndrome and APDS (92, 93). This may be due to increased p110δ signaling, and decreased p110α signaling caused by decreased stability of p110α. Activating point mutations in the iSH2 domain of *PIK3R1* at the C2–iSH2 interface (N564K) also cause APDS2 symptoms (86). This mutant is also found in solid tumors, and it appears in certain situations it can drive p110α-mediated oncogenesis or drive p110δ-mediated immunodeficiency. Loss of function mutations in *PIK3R1* also occur in immune disorders, with patients identified with autosomal recessive nonsense mutations in *PIK3R1* (W298*, R301*) leading to agammaglobulinemia, and severe defects in B-cell development (94, 95).

## Conclusion

Tremendous advances in our understanding of PI3K structure, function, and regulation have occurred in the last decade. Detailed cellular and mice studies have revealed unexpected mechanisms of how PI3Ks are activated. The discovery of patients containing PI3K mutations in cancer, developmental disorders, and immunodeficiencies has revealed the key role of these enzymes in disease. PI3K-specific inhibitors have been developed, and the first PI3K inhibitor, selective for p110δ, has entered the clinic for treatment of blood cancers (14, 96), and other *PIK3CD*-specific inhibitors have showed efficacy in the treatment of APDS (97, 98). PI3K inhibitors may also be useful in targeting the tumor microenvironment (99), and in promoting tumor-specific immune responses (100). However, many PI3K inhibitors have failed in clinical trials for cancer, and there is still extensive work that needs to be done to understand PI3K signaling in human disease. For example, why do the same mutations occur in both cancer and immunodeficiencies, what are the other factors that predispose the same mutation toward a particular disease? Continued examination of PI3K signaling will be essential to fully understand its role in human disease and may reveal unexpected paths to novel therapeutic development.

## Author Contributions

All authors contributed to the writing and editing of the manuscript.

## Conflict of Interest Statement

The authors declare that the research was conducted in the absence of any commercial or financial relationships that could be construed as a potential conflict of interest.
