# Supplementary material for: Molecular Mechanisms of Human Disease Mediated by Oncogenic and Primary Immunodeficiency Mutations in Class IA Phosphoinositide 3-Kinases
Source: Front Immunol. 2018 Mar 19;9:575. doi: 10.3389/fimmu.2018.00575 (PMC5868324; doi:10.3389/fimmu.2018.00575)
Supplement: Supplementary file 1 [file table_1.PDF]

PIK3CD				
**Mutation Residue**	**Disease**	**Inheritance**	**Mutation**	**Paper**
81	APDS 1	Dominant	E81K	Takeda et al., 2017
124	APDS 1	Dominant	G124D	Takeda et al., 2017
334	APDS 1	Dominant	N334K	Dulau Florea et al., 2017; Lucas et al., 2014a
405	APDS 1	Dominant	R405C	Rae et al., 2017
416	APDS 1	Dominant	C416R	Crank et al., 2014
525	APDS 1	Dominant	E525A	Tsujita et al., 2016
525	APDS 1	Dominant	E525K	Coulter et al., 2017; Dulau Florea et al., 2017; Lucas et al., 2014a; Teranishi et al., 2017; Tsujita et al., 2016
929	APDS 1	Dominant	R929C	Wentink et al., 2017
1021	APDS 1	Dominant	E1021K	Angulo et al., 2013; Coulter et al., 2017; Crank et al., 2014; Dulau Florea et al., 2017; Hartman et al., 2016; Elgizouli et al., 2016; Liu et al., 2016; Lucas et al., 2014a; Saettini et al., 2017; Tang et al., 2017; Tsujita et al., 2016; Wentink et al., 2017
1025	APDS 1	Dominant	E1025G	Dulau Florea et al., 2017
**PIK3R1**	Cosmic database for oncogenic PIK3R1 mutations			
**Mutation Residue**	**Disease**	**Inheritance**	**Mutation**	**Paper**
298	Agammaglobulinemia	Recessive	W298*	Conley et al., 2012
301	Agammaglobulinemia	Recessive	R301*	Tang et al., 2017
434-475	APDS 2	Dominant	del 434-475	Deau et al., 2015; Hauck et al., 2017; Kuhlen et al., 2016; Lucas et al., 2014b; Petrovski et al., 2016; Wentink et al., 2017
434-475	APDS 2-SHORT	Dominant	del 434-475	Bravo Garcia-Morato et al., 2017; Petrovski et al., 2016
489	SHORT	Dominant	E489K	Thauvin-Robinet et al., 2013
564	APDS 2	Dominant	N564K	Wentink et al., 2017
631	SHORT	Dominant	R631Q	Thauvin-Robinet et al., 2013
636-653*	SHORT	Dominant	653* (N636Tfs*18)	Dyment et al., 2013
643-651*	SHORT	Dominant	651* (D643Dfs*8)	Barcena et al., 2014
649	SHORT	Dominant	R649W	Bárcena et al., 2014; Chudasama et al., 2013; Dyment et al., 2013; Huang-Doran et al., 2016; Klatka et al., 2017; Schroeder et al., 2014; Thauvin-Robinet et al., 2013
539	SHORT	Dominant	I539del	Thauvin-Robinet et al., 2013
653	SHORT	Dominant	K653*	Klatka et al., 2017
657	SHORT	Dominant	Y657*	Dyment et al., 2013; Huang-Doran et al., 2016
**PIK3R2**				
**Mutation Residue**	**Disease**	**Inheritance**	**Mutation**	**Paper**
373	Overgrowth	#	G373R	Mirzaa et al., 2015; Rivière et al., 2012
376	Overgrowth	#	L376G	Mirzaa et al., 2015
401	Overgrowth	*De novo*	L401P	Nakamura et al., 2013
557	Overgrowth	*De novo*	D557H	Terrone et al., 2016

PIK3CA	Cosmic database for oncogenic PIK3CA mutations			
**Mutation Residue**	**Disease**	**Inheritance**	**Mutation**	**Paper**
81	PROS	#	E81K	Kuentz et al., 2017; Rivière et al., 2012
88	PROS	#	R88Q	Rivière et al., 2012
104	PROS	#	P104L	Kuentz et al., 2017
106	PROS	#	G106V	Kuentz et al., 2017
106,108	PROS	#	G106_R108delinsI	Kuentz et al., 2017
110	PROS	#	E110del	Kuentz et al., 2017
115	PROS	#	R115P	Kuentz et al., 2017
118	PROS	#	G118D	Kuentz et al., 2017
364	PROS	#	R364R	Kuentz et al., 2017; Rivière et al., 2012
365	PROS	#	E365K	Kuentz et al., 2017; Rivière et al., 2012
378	PROS	#	C378Y	Kuentz et al., 2017; Rivière et al., 2012
418	PROS	#	E418K	Kuentz et al., 2017
420	PROS	#	C420R	Kuentz et al., 2017
449	PROS	#	P449T	Kuentz et al., 2017
452	PROS	#	E453K	Kuentz et al., 2017
453	PROS	#	E453del	Rivière et al., 2012
471	PROS	#	P471L	Kuentz et al., 2017
542	PROS	#	E542K	Kuentz et al., 2017
542	PROS	#	E542G	Kuentz et al., 2017
544	PROS	#	T544N	Jansen et al., 2015;
545	PROS	#	E545K	Kuentz et al., 2017; Rivière et al., 2012
546	PROS	#	E546K	Kuentz et al., 2017
546	PROS	#	E546R	Kuentz et al., 2017
546	PROS	#	E546H	Kuentz et al., 2017
726	PROS	#	E726K	Kuentz et al., 2017; Rivière et al., 2012
901	PROS	#	C901F	Kuentz et al., 2017
909	PROS	#	F909L	Kuentz et al., 2017
914	PROS	#	G914A	Kuentz et al., 2017
914	PROS	#	G914R	Kuentz et al., 2017; Rivière et al., 2012
1021	PROS	#	Y1021C	Kuentz et al., 2017; Rivière et al., 2012
1021	PROS	#	Y1021H	Kuentz et al., 2017
1025	PROS	#	T1025N	Kuentz et al., 2017
1025	PROS	#	T1025A	Rivière et al., 2012
1035	PROS	#	A1035V	Kuentz et al., 2017; Rivière et al., 2012
1043	PROS	#	M1043I	Kuentz et al., 2017; Rivière et al., 2012
1043	PROS	#	M1043V	Kuentz et al., 2017
1044	PROS	#	N1044K	Kuentz et al., 2017
1047	PROS	#	H1047L	Lindhurst et al., 2012
1047	PROS	#	H1047Q	Kuentz et al., 2017
1047	PROS	#	H1047R	Lindhurst et al., 2012
1047	PROS	#	H1047Y	Kuentz et al., 2017; Rivière et al., 2012
1049	PROS	#	G1049S	Rivière et al., 2012
1050	PROS	#	G1050S	Kuentz et al., 2017

Supplemental Table 1. Mutations in PIK3CD, PIK3R1, PIK3R2, and PIK3CA that lead to APDS, SHORT, Agammaglobulinemia, and Overgrowth syndromes. PROS = PIK3CA Related Overgrowth Syndromes; Dominant = refers to the expected inheritance. Mutations also occur *de novo*. # = mixture of possible inheritance. Mutations are either *de novo* germline, postzygotic somatic mosaic, or inherited from a parent with germline mosaic mutation; * = nonsense mutation; del = deletion of nucleotides composing stated residue; G106_R108delinsI = deletion of 8 nucleotides, insertion of 2 nucleotides resulting in the loss of three amino acid residues in exchange for one (106GNR->106I)
